# Full-length mitochondrial genome of the triton trumpet *Charonia lampas* (Littorinimorpha: Ranellidae)

**DOI:** 10.1080/23802359.2017.1398610

**Published:** 2017-11-06

**Authors:** In-Young Cho, Keun-Yong Kim, Chang Ho Yi, Il Hun Kim, Yun-Hwan Jung, Sung-Jin Hwang, Jinho Bae, Moongeun Yoon, Min-Seop Kim

**Affiliations:** a National Marine Biodiversity Institute of Korea, Janghang-eup, Republic of Korea;; b Department of Genetic Analysis, AquaGenTech Co., Ltd, Busan, Republic of Korea;; c School of Biological Sciences, College of Natural Sciences, Seoul National University, Seoul, Republic of Korea;; d Department of Eco-Biological Science, Woosuk University, Wanju, Republic of Korea;; e Korea Marine Environment Management Corporation, Seoul, Republic of Korea

**Keywords:** *Charonia lampas*, Littorinimorpha, mitochondrial genome, phylogeny, triton trumpet

## Abstract

The full-length mitochondrial genome of the triton trumpet *Charonia lampas* (Linnaeus, 1758) was analyzed by the primer walking method. Its mitogenome is 15,382 bp in length, comprising 13 protein-coding genes, two ribosomal RNA genes, and 22 transfer RNA genes. The gene order of *C. lampas* is congruent with those previously reported for the infraorder Littorinimorpha. This is the first full-length mitogenome sequence for the genus *Charonia*. In the phylogenetic tree, *C. lampas* formed a monophyletic group with the other species of the superfamily Tonnoidea, but did not show the closest phylogenetic relationship to a species from the same family, Ranellidae.

The triton trumpet *Charonia lampas* (Linnaeus, 1758) is a marine gastropod species with a wide distribution, which attracts great scientific interest owing to its large size, predation on starfish, and production of valuable natural products (Iijima and Egami 1971; Morton 2012). This species is registered as endangered and protected by law in South Korea owing to the decline in its occurrence and population size (NIBR 2014).

A specimen of *C. lampas* was collected from Jeju Island in Korea in 2017, after permission from the Ministry of Environment of Korea. The voucher specimen (MABIK MO00163735) was deposited in the collection of the National Marine Biodiversity Institute of Korea (Seochun, South Korea). Genomic DNA was extracted from its foot tissue in accordance with the method reported by Asahida et al. (1996). To amplify the complete mitochondrial genome (mitogenome) sequences, two independent and overlapping PCR runs were conducted with forward and reverse primers designed in this study, and the PCR products were directly sequenced using a set of 24 sequencing primers. The full-length mitochondrial genome sequence was deposited in the GenBank under the accession number MG181942.

All mitogenome sequences of the infraorders Littorinimorpha and Neogastropoda were retrieved from GenBank. They were aligned together with the *C. lampas* sequence analyzed in this study and refined manually to correct obvious misalignments. The nucleotide matrix of 10 protein-coding genes, excluding ambiguously aligned genes (*nad2*, *nad4* and *nad6*), was created with the first and second positions of codon triplets, after excluding the third codon positions. The final matrix consisted of 2722 and 2722 bp for the first and second codon positions of 10 protein-coding genes, respectively. The alignment information is available upon request in FASTA format. Phylogenetic analysis was conducted using RAxML 7.0.4 (Stamatakis 2006) for maximum-likelihood (ML) analysis.

The full-length mitogenome sequence of *C. lampas* is a circular molecule of 15,382 bp in length, consisting of 13 protein-coding genes, two ribosomal RNA genes and 22 transfer RNA genes. All the protein-coding genes begin with an ATG start codon and stop with a TAA stop codon. The gene order of *C. lampas* is congruent with the previously reported mitogenomes of all species belonging to the infraorder Littorinimorpha, except for the species from the family Vermetidae. This is the first report of a full-length mitochondrial genome sequence for the genus *Charonia*.

On the basis of the full-length mitogenome sequence of *C. lampas* analyzed in this study, a phylogenetic tree was reconstructed by the ML method, with the nucleotide sequence matrix from 10 concatenated protein-coding genes (Figure 1). The tree recovered the strongly supported monophyly of all species belonging to the Littorinimorpha and Neogastropoda, with a 100% bootstrap value against the outgroups (i.e. two *Haliotis* species belonging to the Vetigastropoda). Among them, three species belonging to the superfamily Tonnoidea (i.e. *C. lampas*, *Cymatium parthenopeum* and *Galeodea echinophora*) consistently formed a monophyletic group with high statistical support. However, *C. lampas* did not show the closest phylogenetic relationship to *C. parthenopeum* from the same family, Ranellidae.

**Figure 1.**
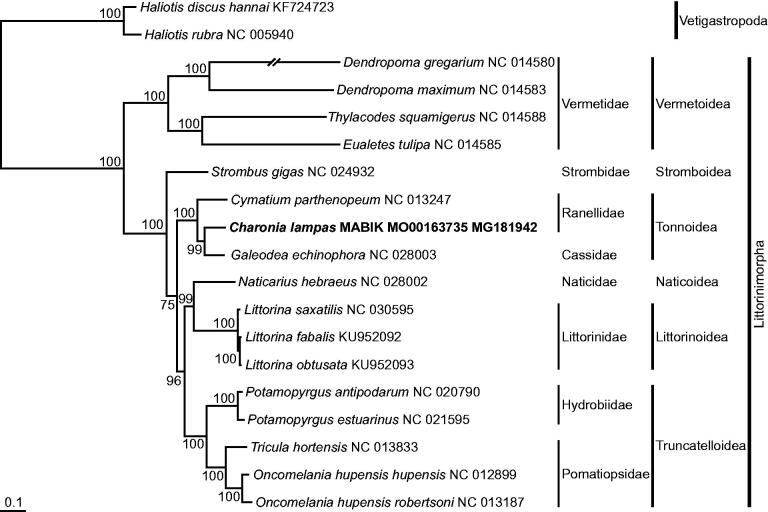
Maximum-likelihood phylogeny based on 10 protein-coding genes in the full-length mitochondrial genomes from the species belonging to the infraorders Littorinimorpha and Neogastropoda. The matrix included the first and second codon positions of 10 protein-coding genes. Two Haliotis species belonging to the Vetigastropoda were used as outgroups. A bootstrap value above 50% in the ML analysis is indicated at each node. The nerite *C. lampas* investigated in this study is shown in bold.
